# Supplementary material for: Longitudinal observational (single cohort) study on the causes of trypanocide failure in cases of African animal trypanosomosis in cattle near wildlife protected areas of Northern Tanzania
Source: PLoS Negl Trop Dis. 2025 Jan 21;19(1):e0012541. doi: 10.1371/journal.pntd.0012541 (PMC11785308; doi:10.1371/journal.pntd.0012541)
Supplement: S1 Box — (DOCX) [file pntd.0012541.s001.docx]

**Box 1**. Variable Definitions of data collected using KoBoCollect

- **Animal weight** was estimated using girth length as described by Lesosky et al. [46].
- **Trypanocide dose** in milligrams was calculated based on drug quantity administered, drug brand and manufacturer’s guidelines.
- Trypanocide **dose description** was categorised as appropriate, underdose or overdose, based on animal weight, dose in milligrams and manufacturer’s instructions. The appropriate dosage range for treatment with ISM, DA and HM was 0.25 - 1 mg/kg, 3.5 - 7 mg/kg and 1 – 1.5 mg/kg, respectively. The appropriate dosage range to provide ISM prophylactic protection was 0.5 - 1 mg/kg. Trypanocide dose description was further categorised as correct, if an appropriate dose or an overdose was administered, or incorrect, if an underdose was given, when assessing the contribution to treatment or prophylactic failure.
- The **reason for treatment**, as reported by farmers, was used to categorise administrations as symptomatic or prophylactic. Symptomatic treatments were for animals that were believed to be suffering from AAT, while prophylactic administrations were given to healthy animals as a preventive measure.
- The **route of administration** was classified as correct in case of the farmer’s reported intention to provide an intramuscular injection (the route described in product data sheets for ISM, DA and HM in cattle), incorrect for any other intended route (e.g., subcutaneous or intravenous).
- Based on the field veterinarian’s evaluation of the farmer’s **competency** to inject trypanocide using the intended route, administrations were classified as competent or incompetent.
- The farmer’s recount of drug storage conditions, and drug manufacturer’s guidelines were considered to determine if **trypanocide storage** was appropriate or inappropriate.
- **Treatment adequacy** was assessed based on trypanocide dose description, route of administration, competency of administration and trypanocide storage conditions. A trypanocide administration was deemed adequate if the following conditions were all met: the drug was not underdosed; the route of administration was intramuscular; the farmer injected the drug competently; the drug storage conditions were in line with the manufacturer’s recommendations.
