# Supplementary material for: Longitudinal observational (single cohort) study on the causes of trypanocide failure in cases of African animal trypanosomosis in cattle near wildlife protected areas of Northern Tanzania
Source: PLoS Negl Trop Dis. 2025 Jan 21;19(1):e0012541. doi: 10.1371/journal.pntd.0012541 (PMC11785308; doi:10.1371/journal.pntd.0012541)
Supplement: S2 Table — (DOCX) [file pntd.0012541.s003.docx]

**Supplementary table 2**. Assumptions and limitations of DA and HM outcome pathways.

| **Pathway** | **Assumptions** | **Limitations and uncertainties** | **Number of cases - DA** | **Number of cases - HM** |
| --- | --- | --- | --- | --- |
| 1 | Since both TX and FU1 samples are PCR negative, it is assumed that the animal is not infected at the time of treatment. | Parasitaemia might fall below the PCR detection threshold during both TX and FU1. | 63 | 3 |
| 2 | At TX, the animal infected with Trypanosoma can test negative due to a low parasitaemia level. The fact that both samples collected at FU1 and FU2 test positive for the same Trypanosoma species, indicates treatment failure. | The animal could be free of infection at TX and become infected over the following week. On the other hand, the animal might test negative at TX due to a low parasitaemia level, turn positive at FU1 because of the release and persistence of parasitic DNA in the bloodstream following parasite death, and subsequently test positive at FU2 due to reinfection. | 0 | 0 |
| 3 | At TX, the animal infected with Trypanosoma can test negative due to a low parasitaemia level. However, at FU1 the animal could test positive due to the release and persistence of parasitic DNA in the bloodstream after parasite death. Given the negative results obtained from the FU2 sample, the treatment is considered to be successful. | The animal could be free of infection at TX and become infected over the following week. Parasitaemia might fall below the PCR detection threshold during FU2. | 7 | 1 |
| 4 | The available evidence does not provide clarity regarding the treatment's outcome. It is assumed that during TX, the animal is infected with Trypanosoma, yet the PCR result is negative due to a low parasitaemia level. The positive FU1 sample could be interpreted as a sign of treatment failure, or potentially a false positive arising from the release and permanence of parasitic DNA in the bloodstream after parasite death.  During FU2, the animal tests positive for a different Trypanosoma species than the one detected at FU1. This occurrence may indicate a reinfection, supporting the hypothesis of a successful treatment. It could be argued, however, that the animal originally hosted two different Trypanosoma species, and the one identified at FU2 was concealed at FU1 due to a low parasitaemia level. In this latter situation, the treatment's outcome is considered to be a failure. | The animal could be free of infection at TX and become infected over the following week. | 1 | 1 |
| 5 | The available evidence does not provide clarity regarding the treatment's outcome. The positive FU1 sample could be interpreted as a sign of treatment failure, or potentially a false positive arising from the release and permanence of parasitic DNA in the bloodstream after parasite death.  During FU2, the animal tests positive for a different Trypanosoma species than the one detected at FU1. This occurrence may indicate a reinfection, supporting the hypothesis of a successful treatment. It could be argued, however, that the animal originally hosted two different Trypanosoma species, and the one identified at FU2 was concealed at FU1 due to a low parasitaemia level. In this latter situation, the treatment's outcome is considered to be a failure.  Varying levels of parasitaemia can lead to PCR negative samples at FU3 and FU4. Prophylactic protection is assumed to be absent. | The animal can be successfully treated and become infected with the same Trypanosoma species over the following week. | 0 | 0 |
| 6 | The negative results from the sample collected during FU1 indicate a successful treatment. This hypothesis is further supported by the evidence of reinfection with a different Trypanosoma species at FU2. | During FU1 and FU2, the animal can test negative for the Trypanosoma species detected at TX due to a low parasitaemia level. In this case, the treatment outcome would be failure. | 1 | 0 |
| 7 | The presence of the same Trypanosoma species in both TX and FU2 samples indicates a treatment failure. The sample obtained at FU1 may be either positive or negative, depending on parasitaemia levels. | The animal can be successfully treated and become infected with the same Trypanosoma species over the following week. Additionally, at FU1 the animal could test positive due to the release and persistence of parasitic DNA in the bloodstream after parasite death. The positive sample collected during FU2 could be indicative of a new case of infection following a successful treatment. | 3 | 1 |
| 8 | The sample collected during FU2 tests negative, suggesting the treatment is successful. During FU1, the animal's sample could be either negative or positive, possibly due to the persistence of parasitic DNA in the bloodstream following parasite death. | Parasitaemia might fall below the PCR detection threshold during FU1 and FU2. | 35 | 1 |
