# Supplementary material for: Longitudinal observational (single cohort) study on the causes of trypanocide failure in cases of African animal trypanosomosis in cattle near wildlife protected areas of Northern Tanzania
Source: PLoS Negl Trop Dis. 2025 Jan 21;19(1):e0012541. doi: 10.1371/journal.pntd.0012541 (PMC11785308; doi:10.1371/journal.pntd.0012541)
Supplement: S3 Table — (DOCX) [file pntd.0012541.s004.docx]

**Supplementary table 3**. Summary of baseline data collected during farms and cattle enrolment in the study.

| **Number of farms enrolled** | 21 |
| --- | --- |
| **Number of cows enrolled** | 630 |
| **Herd size (n cows)** | Min: 33 Max: 603 Mean: 158 Median: 110 |
| **Number of female cows enrolled** | 475/630 (75%) |
| **Weight of female cows (kg)** | Min: 54 Max: 365 Mean: 177 Median: 180 |
| **Age of female cows in years** | Min: 0.5 Max: 12 Mean: 3.5 Median: 3 |
| **Number of male cows enrolled** | 155/630 (25%) |
| **Weight of male cows (kg)** | Min: 48 Max: 641 Mean: 161 Median: 138 |
| **Age of male cows in years** | Min: 0.5 Max: 4.5 Mean: 1.74 Median: 1.5 |
| **Number of farmers that reported seeing tsetse flies in proximity of the herd during the 30 days prior to the baseline visit** | 18/21 |
| **Number of farmers that reported seeing wildlife in proximity of the herd during the 30 days prior to the baseline visit** | 18/21 |
| **Average costs of trypanocides as estimated from a 250kg cow at doses farmers were applying.** | 0.9 USD for ISM  0.3 USD for DA  0.8 USD for HM |
