# Supplementary material for: Longitudinal observational (single cohort) study on the causes of trypanocide failure in cases of African animal trypanosomosis in cattle near wildlife protected areas of Northern Tanzania
Source: PLoS Negl Trop Dis. 2025 Jan 21;19(1):e0012541. doi: 10.1371/journal.pntd.0012541 (PMC11785308; doi:10.1371/journal.pntd.0012541)
Supplement: S4 Table — Samples positive for more than one Trypanosoma species are reported as positive in each relevant column. (DOCX) [file pntd.0012541.s005.docx]

**Supplementary table 4**. Frequency of detection of *T. brucei*, *T. congolense* and *T. vivax* in cattle blood samples at different time points. Samples positive for more than one Trypanosoma species are reported as positive in each relevant column.

|  | **Samples positive for *T. brucei*** | **Samples positive for *T. congolense*** | **Samples positive for *T. vivax*** | **Total samples tested** |
| --- | --- | --- | --- | --- |
| **TX** | 7% (18/266) | 19% (50/266) | 9% (25/266) | 266 |
| **FU1** | 5% (12/260) | 4% (11/260) | 8% (20/260) | 260 |
| **FU2** | 3% (7/229) | 6% (14/229) | 4% (9/229) | 229 |
| **FU3** | 10% (14/140) | 11% (15/140) | 1% (1/140) | 140 |
| **FU4** | 14% (19/139) | 5% (7/139) | 5% (7/139) | 139 |
| **Any time point** | 7% (70/1034) | 9% (97/1034) | 6% (62/1034) | 1034 |
