# Supplementary material for: Longitudinal observational (single cohort) study on the causes of trypanocide failure in cases of African animal trypanosomosis in cattle near wildlife protected areas of Northern Tanzania
Source: PLoS Negl Trop Dis. 2025 Jan 21;19(1):e0012541. doi: 10.1371/journal.pntd.0012541 (PMC11785308; doi:10.1371/journal.pntd.0012541)
Supplement: S5 Table — (DOCX) [file pntd.0012541.s006.docx]

**Supplementary table 5.** Summary of the use of trypanocide brands administered to the cattle enrolled in this study.

| **Trypanocide** | **Recommended dosage range (mg/kg)** | **Brand** | **Continent of origin** | **Average dosage administered (mg/kg)** | **Frequency of use n/317** | **Frequency of IM administration** |
| --- | --- | --- | --- | --- | --- | --- |
| **DA** | **3.5 - 7** | **DA-A** | **Asia** | 6 | 14 (4.4%) | 12/14 (85.7%) |
|  |  | **DA-C** | **Asia** | 5.3 | 21 (6.6%) | 20/21 (95.2%) |
|  |  | **DA-D** | **Europe** | 5.4 | 37 (11.7%) | 34/37 (91.9%) |
|  |  | **DA-E** | **Europe** | 2.3 | 18 (5.7%) | 16/18 (88.9%) |
|  |  | **DA-F** | **Europe** | 6 | 33 (10.4%) | 26/33 (78.8%) |
| **HM** | **1 - 1.5** | **HM** | **Asia** | 1.3 | 20 (6.3%) | 20/20 (100%) |
| **ISM** | **0.25 - 1 (treatment) 0.5 - 1 (prophylaxis)** | **ISM-A** | **Europe** | 0.7 | 83 (26.2%) | 79/83 (95.2%) |
|  |  | **ISM-B** | **Europe** | 0.6 | 78 (24.6%) | 68/78 (87.2%) |
|  |  | **ISM-C** | **Asia** | 0.8 | 13 (4.1%) | 7/13 (53.8%) |
