# Supplementary material for: Longitudinal observational (single cohort) study on the causes of trypanocide failure in cases of African animal trypanosomosis in cattle near wildlife protected areas of Northern Tanzania
Source: PLoS Negl Trop Dis. 2025 Jan 21;19(1):e0012541. doi: 10.1371/journal.pntd.0012541 (PMC11785308; doi:10.1371/journal.pntd.0012541)
Supplement: S6 Table — (DOCX) [file pntd.0012541.s007.docx]

**Supplementary table 6.** Summary of the distribution of hypothesised risk factors for prophylaxis failure in the study dataset.

|  | **Prophylaxis success** | **Prophylaxis failure** |
| --- | --- | --- |
| **Treatment adequacy** |  | |
| Yes | n = 47 | n = 40 |
| No | n = 33 | n = 22 |
| **Brand** |  | |
| Brand A | n = 39 | n = 16 |
| Brand B | n = 37 | n = 37 |
| Brand C | n = 4 | n = 9 |
| **Sex** |  | |
| Sex F | n = 61 | n = 52 |
| Sex M | n = 19 | n = 10 |
| **Age (years)** | Min: 0.5 Max: 10 Mean: 3.5 Median: 3 | Min: 0.5 Max: 10 Mean: 3.3 Median: 2 |
| **Pregnancy status** |  | |
| Positive | n = 25 | n = 13 |
| Negative | n = 55 | n = 49 |
| **Distance from the closest high-density wildlife area (m)** | Min: 0 Max: 20362 Mean: 4576 Median: 3729 | Min: 0 Max: 20362 Mean: 5732 Median: 3729 |
| **Herd size** | Min: 40 Max: 603 Mean: 210 Median: 110 | Min: 40 Max: 603 Mean: 260 Median: 150 |
| **Season** |  | |
| Dry season (Jun-Aug) | n = 45 | n = 37 |
| Long rains season (Mar-May) | n = 10 | n = 6 |
| Short rains season (Sep-Feb) | n = 25 | n = 19 |
