# Supplementary material for: Longitudinal observational (single cohort) study on the causes of trypanocide failure in cases of African animal trypanosomosis in cattle near wildlife protected areas of Northern Tanzania
Source: PLoS Negl Trop Dis. 2025 Jan 21;19(1):e0012541. doi: 10.1371/journal.pntd.0012541 (PMC11785308; doi:10.1371/journal.pntd.0012541)
Supplement: S7 Table — (DOCX) [file pntd.0012541.s008.docx]

**Supplementary Table 7**. Number and proportion of cattle that livestock keepers reported had been treated with Diminazene, Isometamidium or Homidium within the preceding six months in Serengeti District, Tanzania in 2016.

|  | Number | % |
| --- | --- | --- |
| Diminazene | 124/772 | 16.1 |
| Isometamidium | 25/772 | 3.2 |
| Homidium | 37/772 | 4.8 |

Data were collected via questionnaire at an individual animal level at the time of cattle sampling, as part of a randomised cross-sectional survey conducted in Serengeti District, Tanzania, in July-August 2016 and described in Lord et al. 2020.
