# Supplementary figures and images for: Longitudinal observational (single cohort) study on the causes of trypanocide failure in cases of African animal trypanosomosis in cattle near wildlife protected areas of Northern Tanzania
Source: PLoS Negl Trop Dis. 2025 Jan 21;19(1):e0012541. doi: 10.1371/journal.pntd.0012541 (PMC11785308; doi:10.1371/journal.pntd.0012541)

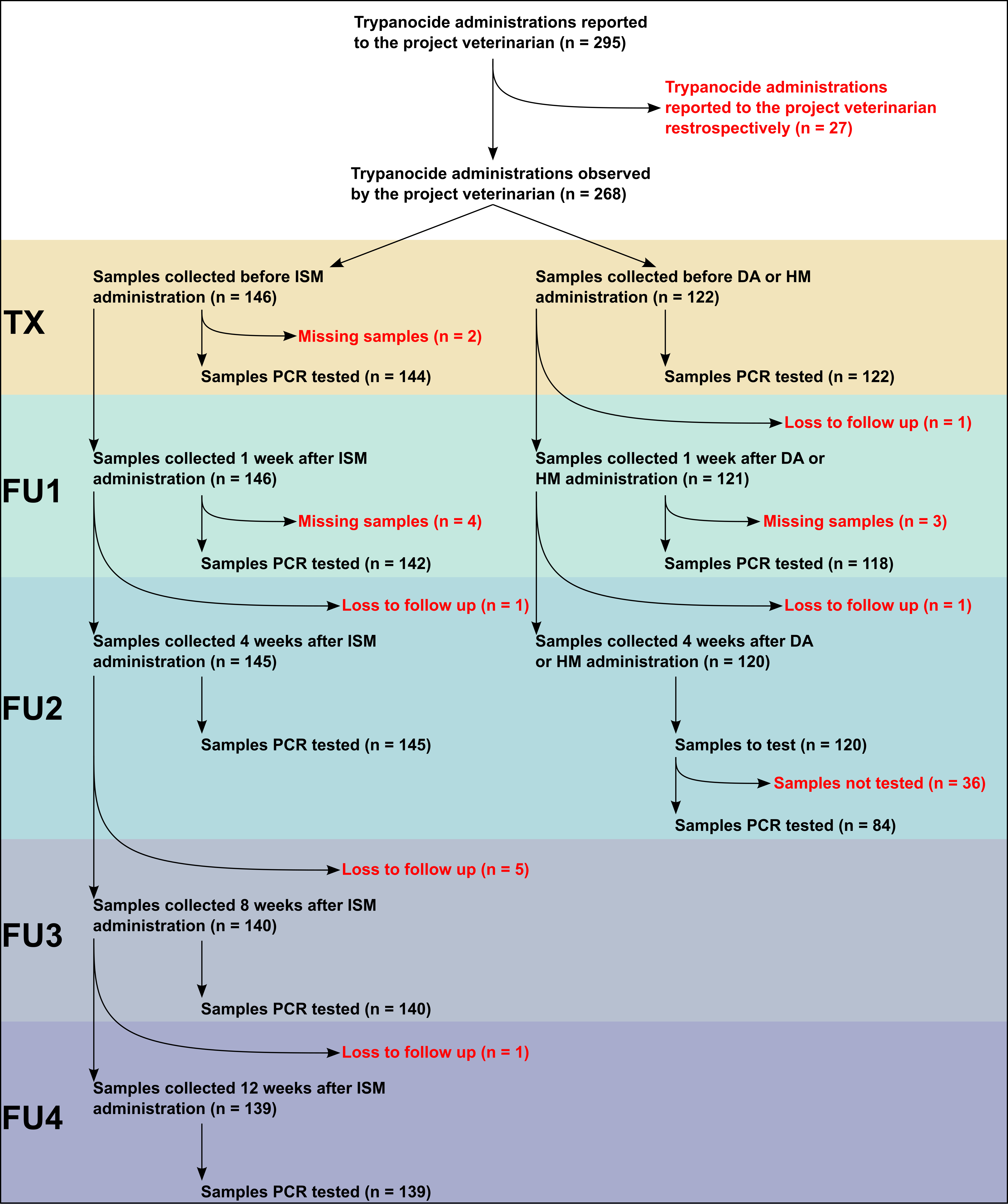

Supplement: S1 Fig — Black text summarises action outcomes, whilst red text provides information on data or sample loss. Abbreviations: TX = day of treatment; FU1 = follow-up one; FU2 = follow-up two; FU3 = follow-up three; FU4 = follow-up four; ISM = isometamidium chloride; DA = diminazine aceturate; HM = homidium chloride. Thirty-six samples collected at FU2 from animals treated with DA or HM were not PCR tested because laboratory results from TX and FU1 samples were sufficient to determine treatment outcome. (PNG) [file pntd.0012541.s009.png]

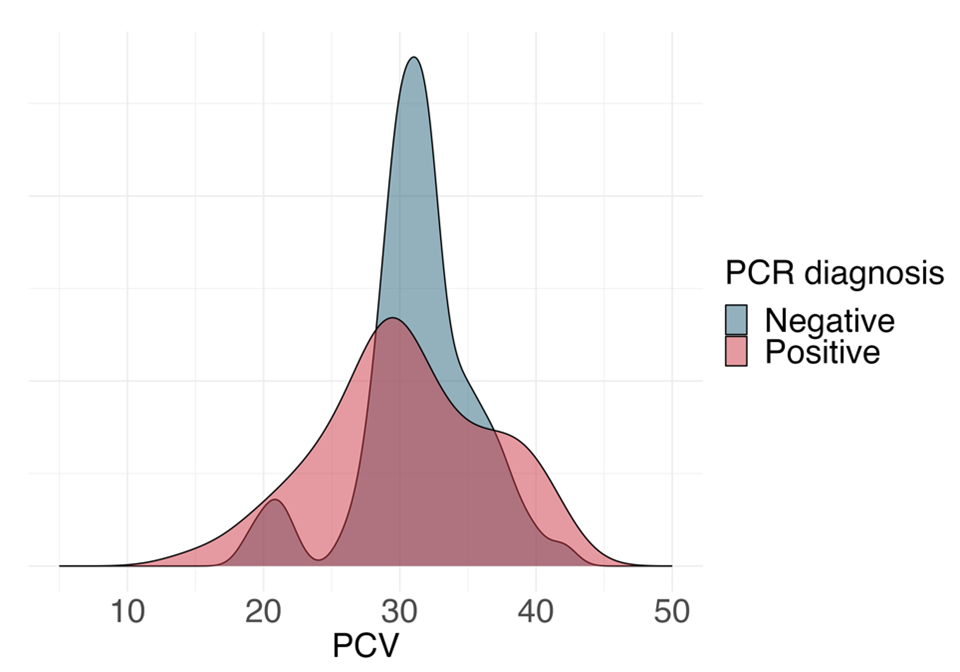

Supplement: S2 Fig — Values derived from samples collected before trypanocide administration are plotted. (PNG) [file pntd.0012541.s010.png]

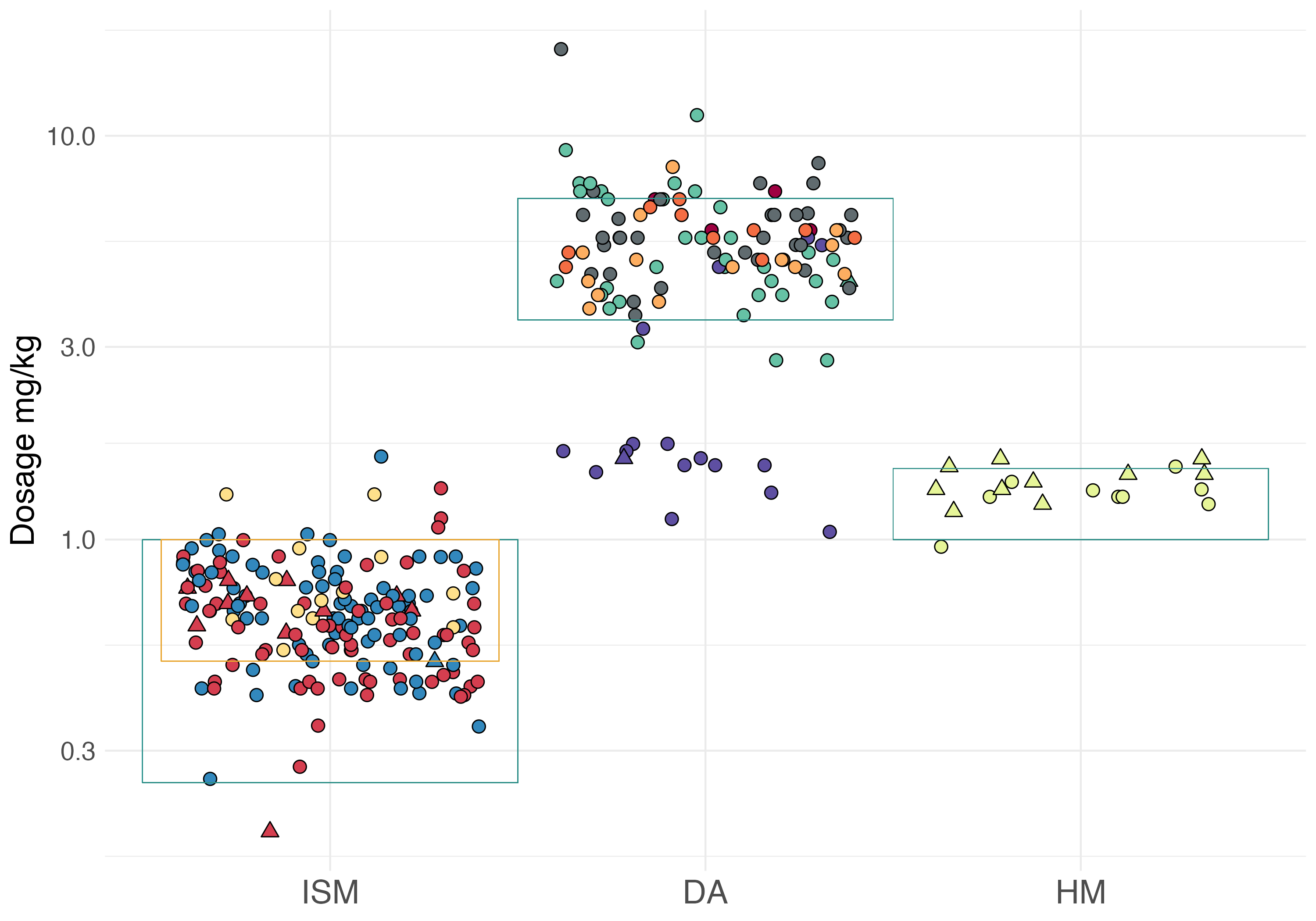

Supplement: S3 Fig — Abbreviations: ISM = isometamidium chloride; DA = diminazine aceturate; HM = homidium chloride. Blue squares represent recommended curative dosages for each trypanocide, while an orange square in the ISM column indicates the recommended prophylactic dosage range. Data points are color-coded by brand. Circular data points indicate a trypanocide administered on its own, while triangles denote a trypanocide given in combination with another one. (PNG) [file pntd.0012541.s011.png]

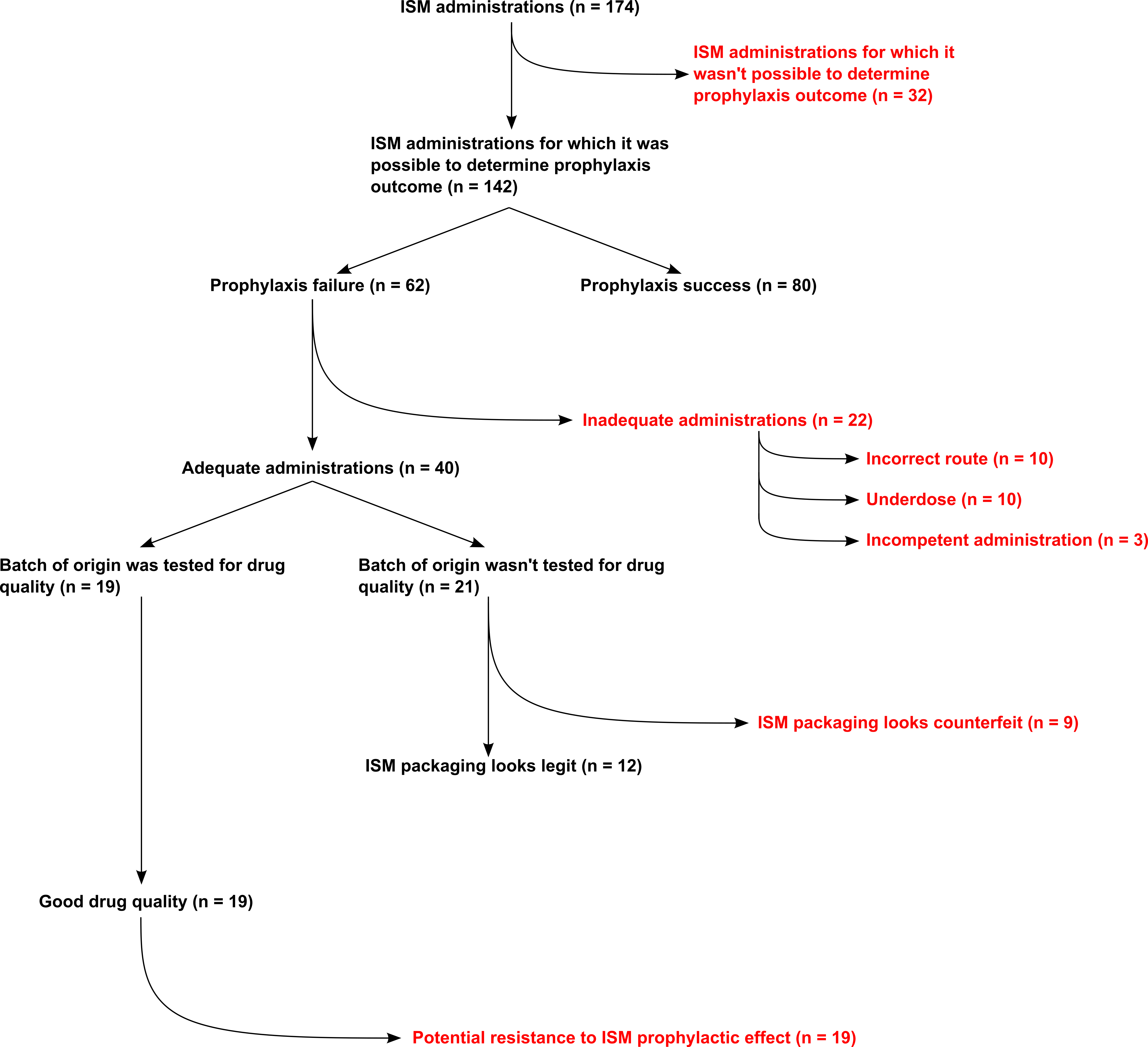

Supplement: S4 Fig — After excluding inadequate administrations (n = 22), untested samples (n = 21), and samples of poor drug quality (n = 0), it is plausible to conclude that some prophylaxis failures may have been due to Trypanosoma strains resistant to ISM prophylaxis. (PNG) [file pntd.0012541.s012.png]
