# Supplementary material for: Longitudinal observational (single cohort) study on the causes of trypanocide failure in cases of African animal trypanosomosis in cattle near wildlife protected areas of Northern Tanzania
Source: PLoS Negl Trop Dis. 2025 Jan 21;19(1):e0012541. doi: 10.1371/journal.pntd.0012541 (PMC11785308; doi:10.1371/journal.pntd.0012541)
Supplement: Attachment — Submitted filename: Response to Reviewers.docx [file pntd.0012541.s014.docx]

Reviewer Comments: PNTD-D-24-01315

Title Longitudinal Observational (single cohort) Study on the Causes of Trypanocide Failure in cases of African Animal Trypanosomosis in Cattle Near Wildlife Protected Areas of Northern Tanzania

**Additional Editor Comments (if provided):**

In addition to the comments from reviewers, I request you to confirm these two minor points.
1. Check graph and number in "Figure 4 : Trypanocide storage conditions" is correctly shown.

*Thank you for this feedback, this was an error and was updated.*

2. Consider result of Fisher's exact test can be explained in the result section, not in the Figure 5.

*This test was removed from the figure and is now in the results section line 385-386*

**Methods**

Reviewer #1: This is a very interesting study on the use of trypanocides in Tanzania, with the objectives clearly articulated. The study design is adequate and flexible enough to accommodate the challenges posed by the study area and the study requirements. The study population is well characterized in an area of "high risk of trypanocide resistance development" was selected based on a perceived high challenge and perceived intense use of trypanocides. From this area, a total of 630 cattle from 21 farms were monitored for a year-long period. This sample size gave enough power for the majority of the required analyses. The statistical analysis was adequate and sufficient to support the conclusions and the study was conducted following high ethical standards.

*Thank you for this feedback.*

Reviewer #2: Lines 280 it should bijou and not bijoux for the bijou bottle

*Thank you, this edit has been, made.*

For figure 2 and the decision tree make the font bigger and easier to read

*Thank you we have oriented this figure to be portrait which allows for larger font and display.*

**Results**

Reviewer #1: The results are clearly presented, making good use of a variety of graphic resources.

Reviewer #2: (No Response)

*Thank you for the feedback on the results*

**Conclusions**

Reviewer #1: The robustness of its experimental design, and chiefly its longitudinal nature, make this study, in a way, a pioneer in the combined study of the complex reasons for failure in the treatment of trypanosome infections, including resistance. This experimental design should be adapted and improved in different ecological niches of sub-Saharan Africa, in order to accurately identify and address the major challenges pertaining the effective use of trypanocides.
The limitations are thoroughly discussed by the authors, as well as the implications of the findings of this work for the treatment of trypanosome infections in Africa. Moreover, the authors established an interesting connection between the failure of the treatment with trypanocides and the reservoir status of cattle for Human African Trypanosomiasis, which points for an important public health dimension.

*Thank you for this positive feedback on our work*

Reviewer #2: The discussion could be streamlined a bit as they are quite some repetitions between the results and discussion and within the discussion itself. It is quite descriptive and long.

*Thank you for this feedback, we feel that we have been as brief as possible in both of these sections given the amount of information which comes from a field trial such as the one we completed. Additionally given the feedback from reviewer 1 we feel the results and discussion can remain in it’s current form.*

**Editorial and Data Presentation Modifications?**

Reviewer #1: The paper is very clear and well written. I added minor comments directly to the manuscript.

Reviewer #2: (No Response)

*Thank you for this feedback on our work.*

**Summary and General Comments**

Reviewer #1: It is a robust, clear and inspiring study.

Reviewer #2: Overall I compliment the authors on this is very interesting piece of work with good methods and interesting results although maybe not as conclusive as one would expect. This is an important and valuable piece of work overall.

*Thank for your feedback on our work*

**Figure resubmission:**

 While revising your submission, please upload your figure files to the Preflight Analysis and Conversion Engine (PACE) digital diagnostic tool, <https://pacev2.apexcovantage.com/>. PACE helps ensure that figures meet PLOS requirements. To use PACE, you must first register as a user. Registration is free. Then, login and navigate to the UPLOAD tab, where you will find detailed instructions on how to use the tool. If you encounter any issues or have any questions when using PACE, please email PLOS at [figures@plos.org](mailto:figures@plos.org). Please note that Supporting Information files do not need this step. If there are other versions of figure files still present in your submission file inventory at resubmission, please replace them with the PACE-processed versions.
